# Public assistance program and depressive symptoms of the recipient: a cross-sectional Japan Gerontological Evaluation Study

**DOI:** 10.1186/s12877-022-02868-0

**Published:** 2022-03-03

**Authors:** Shiho Kino, Daisuke Nishioka, Keiko Ueno, Maho Haseda, Naoki Kondo

**Affiliations:** 1 Department of Social Epidemiology and Global Health, Graduate School of Medicine and School of Public Health, Kyoto University, Floor 2, Science Frontier Laboratory, Yoshida-konoe-cho, Sakyo-ku, Kyoto-shi, Kyoto, 606-8315 Japan; 2 Department of Medical Statistics, Osaka Medical and Pharmaceutical University, Research & Development Center, Daigakumachi 2-7, Takatsuki-shi, Osaka 569-8686 Japan; 3 Department of Health and Social Behavior, Graduate School of Medicine, The University of Tokyo, Hongo 7-3-1, Bunkyo-ku Tokyo, 113-0033 Japan; 4 Institute for Future Initiatives, the University of Tokyo, Hongo 7-3-1, Bunkyo-ku, Tokyo, 113-0033 Japan; 5 Japan Agency for Gerontological Evaluation Study (JAGES Agency), Ueno 6-3-5, Taito-ku, Tokyo, 110-0005 Japan

**Keywords:** Public assistance, Depression, Japan, Aged, Social welfare

## Abstract

**Background:**

Mental health conditions among older recipients of public assistance should be considered because it has been reported that public assistance recipients tend to have higher risks of morbidity than non-recipients, and mental health is strongly related to frailty. We aimed to examine whether older recipients of public assistance were more likely to have depressive symptoms compared to non-recipients.

**Methods:**

Data were obtained from the Japan Gerontological Evaluation Study, a 2016 community-based study of older adults. Poisson regression analyses with a robust error variance using fixed effects were conducted to examine the relationship between receiving public assistance and depressive symptoms controlling for sociodemographic factors. Depressive symptoms were assessed by the Geriatric Depression Scale 15.

**Results:**

We found that the older recipients of public assistance were 1.57 times (95% confidence interval [CI]: 1.47, 1.67) more likely to have depressive symptoms compared to non-recipients. We also found that, when additionally adjusting for indicators of social participation, this relationship was slightly attenuated; however, the recipients still had worse mental health issues (Prevalence ratio: 1.33; 95% CI: 1.25, 1.42).

**Conclusions:**

Even after controlling for sociodemographic factors, older recipients of public assistance tended to be more depressed than non-recipients. However, our findings also indicated that social participation could slightly attenuate the negative relationship between receiving public assistance and depressive symptoms. Therefore, the public assistance program needs to consider the inclusion of mental healthcare support in addition to financial support.

**Supplementary Information:**

The online version contains supplementary material available at 10.1186/s12877-022-02868-0.

## Introduction

Health inequality is one of the major challenges in public health [1]. Targeting specific populations with socioeconomic difficulties has been one of the methods that is used to address health inequality [2]. The public assistance program works as a safety net to ensure the minimum standard of living as well as to promote independence for all citizens living in poverty. Given that the COVID-19 pandemic has led to an increase in the number of people living in poverty, the importance of this program has also increased.

In Japan, eligibility for receiving public assistance is judged by municipal welfare offices. A rigorous means-test for each potential household is conducted by the local municipal welfare office to comprehensively assess their personal assets (i.e., living below the poverty line without assets), ability to work, financial support received from relatives and their use of any other welfare services. In September 2020, 1.63% of Japanese residences had received public assistance [3]. Although the evaluation of the income provided by the recipient varies depending on the household conditions (e.g., the living area, the number of people in the household, and other sources of income among other conditions), this program provides assistance in covering most of the necessities, such as livelihood assistance, education assistance, housing assistance, medical assistance, long-term care assistance, maternity assistance, occupational assistance, and funeral assistance. Thus, one of the specific features of this program is that the recipients are financially protected in addition to receiving free access to healthcare, while non-recipients with the same income need to pay for these necessities. However, recipients of public assistance might be socially isolated and/or exposed to social stigma [4–6]. In fact, internalized welfare stigma, that results from one’s negative feelings regarding being unemployed, has been reported [5]. This mainly occurs because of the financial dependence on taxpayers’ money. Therefore, external stigma that results from the prejudices surrounding receiving public assistance has been reported [6]. For example, people who earn a relatively low income and who use the Supplemental Nutrition Assistance Program (i.e., the food-purchasing assistance program in the U.S.) often feel judged and devalued within their society [7]. In addition, one empirical study reported that unemployed people would attempt to withdraw themselves from their own social networks to cope with the perceived stigma [8]. These facts and experiences lead to the recipients becoming isolated and disconnected from society and could also cause mental health issues.

A review of 32 papers showed that there may be an association between poverty and mental illness [9]. Additionally, a systematic review reported that in high-income countries, recipients of public assistance had worse mental health than non-recipients [10]. However, an empirical study reported that housing assistance was associated with improved health and psychological well-being [11].

A study comprising both a systematic review and meta-analysis reported that there is an evident relationship between depressive symptoms and frailty among older people [12]. Therefore, to aim for healthy aging, addressing mental health among older people is warranted. Some previous studies reported that around 30% of the older Japanese population had depressive symptoms (e.g., Wada et al. [13] reported 33.5% and Sasaki et al. [14] reported between 21.5% and 36.2%). The Japanese government reported that recipients of public assistance were more likely to have a risk of mental health problems than non-recipients [15]. They found that the prevalence of mental health problems was 16.4% among public assistance recipients, while prevalence among non-recipients was 2.5% [15]. In addition, a demographic survey showed that public assistance recipients had a higher risk of carrying out deliberate self-harm behaviors compared to non-recipients [16]. These reports could inform about the importance of public policy targeting mental health service among recipients of public assistance; however, it is not clear whether this difference in mental health is due to the participants’ income levels, or because of other socioeconomic and psychosocial factors associated with being a recipient of a public assistance program (i.e., social isolation and social stigma).

Furthermore, a review study reports that few studies have focused on the health of older public assistance recipients [17]. In Japan, the majority of public assistance recipients are older people (55.5%) [3], and medical assistance consumes the largest portion of the public assistance budget (48.6%) [18]. Furthermore, medical expenditure of hospitalization is significantly higher among the recipients of public assistance compared to patients using universal public health insurance [19], and the recipients of public assistance have a higher risk of frequent outpatient attendance [20]. These studies indicate that other negative factors might be contributing to public assistance recipient’s unhealthy behaviors or health conditions. Therefore, for sustainable public health programs to continue, the role of additional factors that affect the health of older recipients of public assistance must be considered. In this study, we aimed to examine the relationship between receiving public assistance and depressive symptoms among older Japanese adults.

## Methods

### Study population

We used cross-sectional data from a nationwide cohort study in Japan conducted in 2016, called the Japan Gerontological Evaluation Study (JAGES). JAGES aimed to examine social determinants of healthy aging people among the older people by mailing self-reporting questionnaires to approximately 280,000 community-dwelling individuals aged 65 years and older in 39 municipalities. The participants were selected by municipal unit: Randomly selected individuals were included from 22 large municipalities where the population aged 65 years and older was over 5000 people, while all older individuals were included from 17 small municipalities where it was less than 5000 people. The total number of participants was 196,438; the response rate was 70.2%. We excluded individuals who had missing values in the variables used in this study.

### Public assistance status

The participants were asked whether they received public assistance when they answered the questionnaire. The question had three response options: “not receiving public assistance,” “receiving public assistance,” and “applying for public assistance.” Individuals who answered “applying for public assistance” to this question (0.03% of the responses) were excluded from the sample.

### Outcome variables

We used depressive symptoms as an outcome. Depressive symptoms were measured using the Japanese short version of the Geriatric Depression Scale (GDS) with 15 binary questions [21, 22]. The questions included: (1) Are you basically satisfied with your life? (*reverse coded*) (2) Have you dropped many of your activities and interests? (3) Do you feel that your life is empty? (4) Do you often get bored? (5) Are you in good spirits most of the time? (*reverse coded*) (6) Are you afraid that something bad is going to happen to you? (7) Do you feel happy most of the time? (*reverse coded*) (8) Do you often feel helpless? (9) Do you prefer to stay at home, rather than going out and doing new things? (10) Do you feel you have more problems with memory than most other people? (11) Do you think it is wonderful to be alive now? (*reverse coded*) (12) Do you feel pretty worthless the way you are now? (13) Do you feel full of energy? (*reverse coded*) (14) Do you feel that your situation is hopeless? (15) Do you think that most people are better off than you are? Then, the overall summed score (range: 0–15) was dichotomized, and individuals with a score of ≥ 5 were considered to have depressive symptoms. This cutoff point was validated to screen major depressive symptoms with the area under the receiver operating characteristic curve of 0.94 (sensitivity of 92% and specificity of 87%) against the Structured Clinical Interview for the *Diagnostic and Statistical Manual of Mental Disorders, Third Edition, Revised*, as the gold standard [23].

### Explanatory variables

We considered sociodemographic characteristics including age (10-year unit), gender (male vs. female), marital status (unmarried [single, widowed, or divorced] vs. married), and education (9 years or less vs. more than 9 years). Additionally, we controlled for household income (including subsidies based on public assistance and pension; one-million Japanese yen unit), the number of household members, and comorbidity. Comorbidity was assessed by counting the number of the following 16 diagnosed diseases (range: 0–16): hypertension, stroke (e.g., brain hemorrhage), heart disease, diabetes, hyperlipidemia, respiratory disease (e.g., pneumonia, bronchitis), gastrointestinal, liver or gallbladder disease, kidney or prostate gland disease, musculoskeletal disease (e.g., osteoporosis, arthrosis), traumatic injury (e.g., fall, fracture), cancer, blood or immune system disease, dementia (e.g., Alzheimer’s disease), Parkinson’s disease, eye disease, and ear disease.

One of the issues faced by recipients of public assistance is social isolation due to a lack of social interaction [4]. Hence, we included the following four items to measure social interaction and social participation [24, 25]: (1) “How often do you see your friends?” (2) “How many friends/acquaintances have you seen over the past month? Count the same person as one, no matter how many times you have seen him/her.” (3) “How often do you attend activities for a sports club?” (4) “How often do you attend activities for hobby clubs?” Ratings for items one, three, and four ranged from 1 (rarely) to 6 (almost every day). Ratings for item two ranged from 1 (none) to 5 (10 or more). Responses to each item were standardized, resulting in a mean of zero and a standard deviation of one. Higher scores indicate greater social participation.

### Statistical analysis

All analyses were performed using Stata MP16 [26]. Descriptive statistics were used to compare the scores of recipients and non-recipients of public assistance.

We performed a series of Poisson regression analyses with a robust error variance using fixed effects. To adjust the geographical variation, we coded each municipality as a fixed-effect dummy variable. The fixed-effect variable allows us to control for unobserved municipal heterogeneity, such as geographical, cultural, historical, and social conditions at the time of data collection [27]. First, we adjusted only for age and gender (Model 1). In Model 2, education and marital status were further adjusted. We additionally controlled for household income, the number of household members, and comorbidity (Model 3). In the fully adjusted model, the four indicators of social interaction/participation were also included (Model 4). As we included the four indicators simultaneously, we checked variance inflation factors (VIF) for multicollinearity. The VIF was 1.77 for meeting friends, 1.74 for the number of friends, 1.61 for participation in sports clubs, and 1.71 for participation in hobby clubs, in which all the scores were less than 10, suggesting multicollinearity was less likely to be problematic. From each model, prevalence ratios (PR) were presented. The significance level for all statistical analyses was kept at *p* < 0.05.

As a sensitivity analysis, we employed the propensity score matching method to match the conditions between recipients of public assistance and non-recipients. The analytic sample was restricted to those with a household income of less than 400 million Japanese yen for this analysis. This population comprised 68.6% of non-recipients in the survey. In calculating the propensity scores balancing the matching groups, we selected the three variables that are evaluated when considering applications for public assistance in Japan―household income, number of household members, and comorbidity. We used one-to-one caliper (0.2) matching with no replacement, using Stata command “teffect psmatch.” To confirm the matching balance between the two groups, we checked that the standardized differences were lower than 0.1 after matching. Then, we performed a series of Poisson regression analyses with a robust error variance using fixed effects.

## Results

Table 1 shows the characteristics of the study participants. The percentage of valid participants was 47.5% (the percentage of missing values ranged from 8.36% [for age] to 24.78% [for depressive symptoms]; participants who did not report if they were recipients of public assistance accounted for 9.92%). More missing values were found among non-recipients than recipients. Out of 93,280 participants who answered all the variables used for the analyses, 1,093 (1.17%) received public assistance. More than half of the recipients had depressive symptoms, while 21% of non-recipients had depressive symptoms. The group of recipients included more males, less educated individuals, and unmarried people compared to the group of non-recipients. In addition, household income was more than twice among non-recipients. The number of household members was higher (2.62 vs. 1.85), while comorbidity was lower (1.45 vs. 1.65) among non-recipients compared to recipients. In addition, non-recipients had a higher level of social interaction/participation.

**Table 1:** Demographic characteristics of analytic samples

	Recipients of public assistance	Non-recipients of public assistance
	(*n* = 1,093)	(*n* = 92,187)
Depressive symptoms (GDS ≥ 5)	50.14% (*n* = 548)	20.68% (*n* = 19,064)
Age (mean; 10-year unit)	7.29 (SD = 0.58)	7.31 (SD = 0.60)
Sex		
Male	56.82% (*n* = 621)	52.16% (*n* = 48,081)
Female	42.18% (*n* = 472)	47.84% (*n* = 44,106)
Education		
≤ 9 years	42.36% (*n* = 463)	25.90% (*n* = 23,877)
> 9 years	57.64% (*n* = 630)	74.10% (*n* = 68,310)
Marital status		
Unmarried	61.94% (*n* = 677)	22.48% (*n* = 20,728)
Married	38.06% (*n* = 416)	77.52% (*n* = 71,459)
Household income (mean; one-million JPY unit)	1.88 (SD = 2.05)	4.00 (SD = 2.76)
Household number (mean)	1.85 (SD = 1.25)	2.62 (SD = 1.35)
Comorbidity (mean)	1.65 (SD = 1.35)	1.45 (SD = 1.25)
Meeting friends (mean)	3.11 (SD = 1.65)	3.63 (SD = 1.58)
Number of friends (mean)	2.72 (SD = 1.33)	3.50 (SD = 1.36)
Participation in sports clubs (mean)	1.45 (SD = 1.20)	2.14 (SD = 1.71)
Participation in hobby clubs (mean)	1.55 (SD = 1.21)	2.23 (SE = 1.60)

*SD* standard deviation

Table 2 shows the results of the Poisson regression with a robust error variance using fixed effects to examine the relationship between depressive symptoms and receiving public assistance. The recipients of public assistance were about twice as likely to have depressive symptoms in the model adjusted for age and gender (Model 1) (PR: 2.38; 95% confidence interval [CI]: 2.24, 2.53) and the model further adjusted for marital status and education (Model 2) (PR: 1.91; 95% CI: 1.79, 2.03). Furthermore, in the model additionally adjusted for household income, the number of household members, and comorbidity, the PR slightly dropped to 1.57 (95% CI: 1.47, 1.67) (Model 3). In this fully adjusted model (Model 4), the recipients were 1.33 times (95% CI: 1.25, 1.42) more likely to have depressive symptoms.

**Table 2:** The relationship between depressive symptoms and public assistance from Poisson regression analyses with a robust error variance using fixed effects

	**Model 1**		**Model 2**		**Model 3**		**Model 4**	
	PR	95%CI	PR	95%CI	PR	95%CI	PR	95%CI
Public assistance (ref: no)	**2.38**	(2.24–2.53)	**1.91**	(1.79–2.03)	**1.57**	(1.47–1.67)	**1.33**	(1.25–1.42)
Age (10-year unit)	**1.21**	(1.19–1.24)	**1.10**	(1.07–1.12)	1.01	(0.99–1.03)	0.99	(0.98–1.01)
Sex (ref. male)	**0.97**	(0.95–0.99)	**0.87**	(0.84–0.89)	**0.88**	(0.86–0.90)	1.02	(0.99–1.05)
Education > 9 years (ref: ≤ 9 years)			**0.73**	(0.71–0.75)	**0.81**	(0.78–0.83)	**0.87**	(0.85–0.90)
Marital status (ref. unmarried)			**0.69**	(0.67–0.71)	**0.74**	(0.72–0.76)	**0.76**	(0.74–0.78)
Household income (mean; one-million JPY unit)					**0.89**	(0.88–0.89)	**0.91**	(0.90–0.91)
Household number (mean)					**1.04**	(1.03–1.05)	**1.02**	(1.01–1.03)
Comorbidity (mean)					**1.21**	(1.20–1.22)	**1.19**	(1.18–1.20)
Meeting friends							**0.88**	(0.86–0.89)
Number of friends							**0.81**	(0.80–0.83)
Participation in sports clubs							**0.90**	(0.88–0.92)
Participation in hobby clubs							**0.89**	(0.87–0.90)

*PR* Prevalence ratio

As a sensitivity analysis, we conducted propensity score matching and then analyzed the relationship between receiving public assistance and depressive symptoms. The standardized differences after matching were -0.04 for household income, 0.06 for number of household members, -0.07 for comorbidity, indicating that two groups are balanced after matching. The results from Poisson regression analyses were in line with the main findings (the results are presented in Supplemental Table 1).

## Discussion

This study examined the relationship between receiving public assistance and depressive symptoms among older Japanese people. We found that public assistance recipients were more likely to have depressive symptoms than non-recipients. Furthermore, we found that this negative relationship between public assistance and depressive symptoms could be attenuated by social participation.

While public assistance financially protects people, these findings suggest that recipients of public assistance face other burdens. First, the recipients might have faced social stigma, that could be one of the reasons for the depressive symptoms. The support for them is financed through the nation’s taxes; therefore, some recipients may feel that they live without working (internal stigma) [5] and some non-recipients may feel prejudice against those receiving public assistance (external stigma) [6]. Social stigma results in mental health issues among some recipients. Furthermore, these stigma-based mental health issues may lead to unhealthy behaviors, causing further health problems [28]. Second, they might have already had health issues when they started receiving public assistance. The amount of public assistance a person receives is partially assessed based on their ability to work. Some people may not be able to work due to mental health problems or physical health problems in addition to mental illness. In fact, 25.1% of recipients receive public assistance due to injury or illness [29]. Third, the recipients might be isolated from society, that might have a negative impact on their mental health. Reviews have reported the well-established evidence that social isolation is related to worse health [30–32]. In addition, Fukawa suggested that social participation is essential for recipients to remove social isolation and improve independence [4].

Our findings suggest some policy implications. First, we suggest reinforcing skill training for caseworkers to prevent inappropriate communication from contributing to social stigma and social isolation. Although caseworkers have finished the designated course of social work and are certified by the government, they currently have a heavy workload that may result in difficulties in providing sensitive support for recipients. Second, the regular visits made by the caseworkers to the recipients’ residence can be further used; caseworkers can connect the recipients with other health professionals, such as public health nurses, psychiatric social workers, or clinical psychologists, if they notice issues related to their mental health during the visits. This is because the primary focus of caseworkers is on providing support for employment, not on monitoring health concerns. Meanwhile, it is also important to train the supporters of public assistance recipients to be aware of their implicit bias and prejudice that could lead to stigma, in order to mitigate it [33].Third, from July 2020, the Social Welfare Act added the reinforcement of community support with financial support for public assistance recipients [34]. This act aims to support people who suffer from poverty, providing comprehensive, individual, early, continuous, decentralized, and creative support, to secure independence and dignity and create a supportive community. In addition, from January 2021, health management support for recipients of public assistance by welfare workers is mandatory by law for welfare offices of municipalities that provide public assistance [35, 36]. Taking advantage of these initiatives, we suggest strengthening partnerships between medical and social care providers and informal and formal community programs that can alleviate the problems of isolation and stigma (e.g., through social prescribing activities) [37].

This study would be the first study to present depressive symptoms among public assistance recipients in the older Japanese population, though there are some limitations. As this is a cross-sectional study, we could not establish temporality, that may lead to reverse causation. For example, depressive symptoms might be a reason for reluctant social participation. Further longitudinal research studies are needed to examine the differences in depressive symptoms before and after receiving public assistance programs. In addition, we could not consider personal assets in the analysis, being an important factor for receiving public assistance. In particular, if non-recipients have assets other than income, our findings might be underestimated. However, it is difficult to compare possession of assets and spending on necessities, including medical costs. This suggests that future studies should consider personal assets. In addition, although we have discussed that stigma might play a role in mental health among older recipients of public assistance, we could not examine the influence of stigma in the analyses. Therefore, future studies need to consider the impact of stigma when examining the recipients’ mental health. Furthermore, the valid participants were 47.5% of the total participants, which might cause a selection bias. Lastly, even though the number of comorbidities between the recipients and non-recipients was matched, the severity of health problems could not be considered.

## Conclusions

In conclusion, we found that older recipients of public assistance in Japan were more likely to have depressive symptoms than non-recipients. However, it was also indicated that social participation could slightly attenuate the negative relationship between receiving public assistance and depressive symptoms. Our findings suggest that the public assistance program would need to consider the inclusion of mental health support and community support in addition to financial support. Future studies should explore the role of community and social characteristics that can potentially mitigate or strengthen the non-financial risk factors for mental health among public assistance recipients, such as social capital and social stigma.

## Supplementary Information

**Additional file 1: Table 1. **Results from Poisson regression analyses with a robust error variance using fixed effects after propensity score matching.

## Data Availability

The data underlying this study are from the JAGES and contain sensitive information. The data for research purposes are available upon request. Requests for the data can be made to dataadmin.ml@jages.net.

## Abbreviations

GDS: Geriatric Depression Scale 15
PR: Prevalence ratio
JAGES: Japan Gerontological Evaluation Study
JPY: Japanese Yen
SD: Standard deviation
CI: Confidence interval
VIF: Variation inflation factors
