# Supplementary material for: Public assistance program and depressive symptoms of the recipient: a cross-sectional Japan Gerontological Evaluation Study
Source: BMC Geriatr. 2022 Mar 3;22:177. doi: 10.1186/s12877-022-02868-0 (PMC8890952; doi:10.1186/s12877-022-02868-0)
Supplement: Supplementary file 1 — Additional file 1: Table 1. Results from Poisson regression analyses with a robust error variance using fixed effects after propensity score matching. [file 12877_2022_2868_MOESM1_ESM.docx]

**Supplemental Table 1.** Results from Poisson regression analyses with a robust error variance using fixed effects after propensity score matching

|  | **Model 0** | | **Model 1** | | **Model 2** | | **Model 3** | |
| --- | --- | --- | --- | --- | --- | --- | --- | --- |
|  | PR | 95%CI | PR | 95%CI | PR | 95%CI | PR | 95%CI |
| Public assistance (ref: no) | **1.85** | (1.31–2.61) | **1.73** | (1.23–2.43) | **1.72** | (1.22–2.42) | **1.56** | (1.12–2.16) |
| Age (10-year unit) |  |  | **1.13** | (1.03–1.23) | **1.13** | (1.03–1.23) | **1.12** | (1.03–1.22) |
| Sex (ref. male) |  |  | **0.76** | (0.68–0.85) | **0.75** | (0.67–0.84) | **0.85** | (0.76–0.95) |
| Education >9 years (ref: ≤9 years) |  |  |  |  | 0.99 | (0.89–1.10) | 0.98 | (0.88–1.08) |
| Marital status (ref. unmarried) |  |  |  |  | 0.95 | (0.82–1.09) | **0.88** | (0.76–1.01) |
| Meeting friends |  |  |  |  |  |  | **0.86** | (0.80–0.93) |
| Number of friends |  |  |  |  |  |  | **0.92** | (0.85–0.99) |
| Participation in sports clubs |  |  |  |  |  |  | 0.99 | (0.88–1.11) |
| Participation in hobby clubs |  |  |  |  |  |  | **0.71** | (0.63–0.80) |

PR: Prevalence ratio
